# If You Know Them, You Avoid Them: The Imperative Need to Improve the Narrative Regarding Perioperative Adverse Events

**DOI:** 10.3390/jcm11174978

**Published:** 2022-08-25

**Authors:** Michael Eppler, Aref S. Sayegh, Mitchell Goldenberg, Tamir Sholklapper, Sij Hemal, Giovanni E. Cacciamani

**Affiliations:** 1 USC Institute of Urology and Catherine and Joseph Aresty Department of Urology, University of Southern California, Los Angeles, CA 90033, USA; 2 Department of Urology, Einstein Healthcare Network, Philadelphia, PA 19141, USA

There are few things in life as exciting as growing up in the countryside. As adults, we can reminisce on the things we take for granted as children: clean air, seemingly limitless space for playing with friends, and, of course, direct interaction with nature. Many of us remember a sense of awe in seeing fireflies appear around us on warm summer nights. Some may even remember the challenge of catching these fireflies so that we could further admire these unique creatures. However, those who successfully caught them likely learned an essential life lesson—to study these creatures, you need the proper tool to catch them first. The appropriate net made all the difference, with wide enough holes to allow air to pass yet narrow enough to keep the fireflies from flying away.

Similarly, we have long sought to evaluate the appropriate variables in evidence-based medicine, using the correct “catching” tools—capturing data associated with patient outcomes and avoiding searching for the red-herring incident findings. These tools have become increasingly crucial in the surgical literature, as we have seen a rapid expansion in a range of research efforts, ranging from small case series to big data analytics and meta-analyses of patient outcomes. Surgical and anesthesiologic outcomes are no exception. As a medical community, we are still evolving to find commonly shared tools to capture relevant aspects of surgery that impact patient outcomes, such as adverse events (AEs).

To comprehensively understand AEs, we need first to devise tools to collect and report them in a standardized fashion and capture any aspect of them.

**Figure 1:**
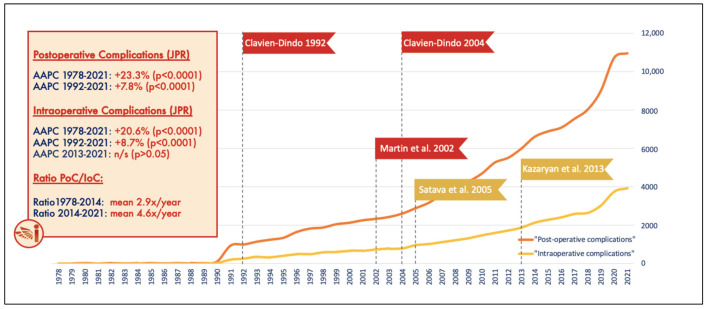
Trends over time of articles reporting “postoperative complications” (orange line) and “intraoperative complications” (yellow line) as one of the outcomes of interest, by year. The join point regression (JPR) analysis was performed to evaluate the trends over time. Average annual percent change (AAPC) is reported to describe increasing or decreasing trends within the search period (1978–2021). PoC: postoperative complication; IoC: intraoperative complication; N/S: not significant; ar: article [1,2,3,8,24].

The reporting of postoperative AEs has already been established by both the Martin criteria and the Clavien–Dindo classification system [1,2]. Postoperative complications (“*Postoperative Complication*” search term in Web of Science) have also been reported more as an outcome of interest (Figure 1). Its standardization and widespread adoption have likely enabled the research community to better understand the underlying causes and prevent complications during the postoperative course across most surgical specialties. 

The standardized assessment of intraoperative adverse events (iAEs) has not yet hit the mainstream despite efforts defining “surgical errors” [3] and proposals for grading systems [4,5,6,7,8]. When compared to the Clavien–Dindo classification system, iAE severity systems are used less [9]. The Intraoperative Complication Assessment and Reporting with Universal Standards (ICARUS) Global Surgical Collaboration is devoted to bringing iAE assessment, grading, and reporting to the mainstream [9,10,11,12,13].

The fact that postoperative AEs are well-studied [14,15,16,17,18,19,20,21] should encourage the surgical community that it is possible to do the same for iAEs. Postoperative AE reporting and grading did not enter the mainstream by chance; there is now widespread acceptance by the medical community, but its utilization started slow [1,2,22,23,24].

It is vital to identify and grade iAEs, as they can be associated with increased patient morbidity and post-operative complication rates [7,25]. For this reason, the surgical/interventional and anesthesiologic community needs a comprehensive “eco-system” to reliably report and grade iAEs.

Explanations for its underutilization are a fear of litigation, emotional toll, and lack of standardization of cross-specialty AE definitions [26]. To reinforce this last point, at the present moment, there are over a dozen published definitions of surgical error [27], and even more definitions for AEs [28], complicating any attempt for large-scale analysis. The definition of iAEs is historically thought of as any deviation from the surgical course that could result in patient harm [28], but likely requires modification to truly capture the many nuances of surgery that increase patient risk.

If we have knowledge of the possible AEs that can occur during the time in the operating room (OR) and contribute to worse patient outcomes, we can potentially avoid them. **If you know them, you avoid them.** Every medical student learns of the triangle of doom in hernia repair. This is a well-studied, easily identifiable step that when paid attention to, can prevent a life-threatening event. Surgery and anesthesia during the surgical/interventional procedures are complex processes, requiring seamless coordination and collaboration between surgeons, anesthesiologists, and nurses, with many less obvious steps that can go wrong. If we can standardize data collection and devise methods for large-scale analysis, while demonstrating its importance to the surgical community, we can uncover many hidden triangles of doom that once known, might reduce AE incidence and improve outcomes and patient safety through medical education.
